# The Current State of Beta-Cell-Mass PET Imaging for Diabetes Research and Therapies

**DOI:** 10.3390/biomedicines9121824

**Published:** 2021-12-03

**Authors:** Pierre Cheung, Olof Eriksson

**Affiliations:** Science for Life Laboratory, Department of Medicinal Chemistry, Uppsala University, SE-75183 Uppsala, Sweden; olof.eriksson@ilk.uu.se

**Keywords:** beta-cell mass, diabetes, positron emission tomography

## Abstract

Diabetes is a chronic metabolic disease affecting over 400 million people worldwide and one of the leading causes of death, especially in developing nations. The disease is characterized by chronic hyperglycemia, caused by defects in the insulin secretion or action pathway. Current diagnostic methods measure metabolic byproducts of the disease such as glucose level, glycated hemoglobin (HbA1c), insulin or C-peptide levels, which are indicators of the beta-cell function. However, they inaccurately reflect the disease progression and provide poor longitudinal information. Beta-cell mass has been suggested as an alternative approach to study disease progression in correlation to beta-cell function, as it behaves differently in the diabetes physiopathology. Study of the beta-cell mass, however, requires highly invasive and potentially harmful procedures such as pancreatic biopsies, making diagnosis and monitoring of the disease tedious. Nuclear medical imaging techniques using radiation emitting tracers have been suggested as strong non-invasive tools for beta-cell mass. A highly sensitive and high-resolution technique, such as positron emission tomography, provides an ideal solution for the visualization of beta-cell mass, which is particularly essential for better characterization of a disease such as diabetes, and for estimating treatment effects towards regeneration of the beta-cell mass. Development of novel, validated biomarkers that are aimed at beta-cell mass imaging are thus highly necessary and would contribute to invaluable breakthroughs in the field of diabetes research and therapies. This review aims to describe the various biomarkers and radioactive probes currently available for positron emission tomography imaging of beta-cell mass, as well as highlight the need for precise quantification and visualization of the beta-cell mass for designing new therapy strategies and monitoring changes in the beta-cell mass during the progression of diabetes.

## 1. Human Pancreas

The human pancreas is an elongated organ situated in the left hypochondriac and epigastric region of the abdomen with a dual exocrine and endocrine function. The majority of the pancreas is composed of exocrine cells (~98–99%), which are organized into acini, that release a mixture of digestive enzymes and bicarbonate to help with digestion. On the other hand, the endocrine cells (~1–2%) are organized into clusters named islets of Langerhans and dispatched heterogeneously over the pancreas. They are divided into five different cell types (alpha, beta, delta, PP, epsilon), which were discovered through histochemical and immuno-staining. Among them, beta cells account for ~50–80% of the pancreatic endocrine cell population and are responsible for insulin production. Insulin is a hypoglycemic peptide hormone that plays a major role in maintaining proper glucose metabolism and is tightly linked to pathologies such as diabetes mellitus [1].

## 2. Diabetes Mellitus

Diabetes mellitus also termed “sweet diabetes” or simply diabetes, is a chronic metabolic disease affecting over 400 million people worldwide, and it is one of the leading causes of death, especially in developing nations. Diabetes is characterized by chronic hyperglycemia caused by defects in the insulin secretion or action pathway. Common symptoms of diabetes induced hyperglycemia are polyuria (excessive urine production) accompanied by polydipsia (excessive thirst), with long term complications of the disease including ulcers, retinopathy and neuropathy [2]. As diabetes etiology is fairly diverse, the disease has been classified into various subtypes. A non-exhaustive list of them include the following: monogenic defects, gestational, type 1 and type 2 [3].

### 2.1. Monogenic Diabetes

The monogenic form of diabetes is associated with a single gene defect, most often related to insulin secretion pathways. The neonatal form, which includes patients under the age of 6 months, is associated with many methylation abnormalities on the chromosome 6q24 [4], as well as mutations in pancreatic ATP-sensitive potassium channels that are responsible for insulin release [5]. Maturity-onset diabetes of the young (MODY) forms a group comprised of several autosomic dominant disorders that often affect patients under the age of 25. The many MODY variants are defined by their genetic defects and cause symptoms of diabetes with various degrees of severity. Common causes of MODY are genetic defects of GCK (Glucokinase), HNF1A (hepatocyte nuclear factor 1-alpha) and HNF4A (hepatocyte nuclear factor 4-alpha), which are involved in glucose sensing and glycemic regulation [6].

### 2.2. Gestational Diabetes

Gestational diabetes (GDM) is described as the onset of glucose intolerance during pregnancy. This results in significantly increased risks for the mother and fetus of developing diabetes and cardiovascular issues later in life. The incidence of GDM has been increasing through the years with the emergence of a new generation of diabetic childbearing women [7].

### 2.3. Type 1 Diabetes

Type 1 diabetes (T1D) affects ~10% of the diabetic population and is characterized by the destruction of the insulin-producing beta cells in the pancreatic islets by an immune-mediated process, resulting in the disruption of proper glucose regulation. Clear evidence points towards a T-cell-mediated autoimmune process in the destruction of beta-cells [8,9], which is supported by the discovery of several autoantigens, such as GAD65, GAD67 and ICA69, in recent years. Th1 lymphocytes in particular have been demonstrated to be closely associated with T1D pathophysiology through the production of pro-inflammatory cytokines such as IL-4, IL-10 and gamma interferon. Although T-cells have a clear involvement in T1D etiology, B-cells have a less defined role in the development of T1D, with reports suggesting inconsistent humoral activity [10]. Strong genetic risk factors have been identified through associated studies, with strong evidence pointing towards defects in the human leucocyte antigen region (HLA), such as the HLA-DR and HLA-DQ loci [11].

### 2.4. Type 2 Diabetes

The pathophysiology of type 2 diabetes (T2D) is still uncertain; however, it accounts for the majority of diabetes mellitus cases. The main feature that defines type 2 diabetes is insulin resistance, leading to increased insulin production, which can then beget the overload and failure of the pancreatic beta cells. Reactive oxygen species (ROS), for instance, are a byproduct of glucose metabolism from the cellular mitochondria and have been posited to induce beta-cell stress and exhaustion [12]. On the other hand, a high metabolic load can induce the genetic identity loss in beta cells through a dedifferentiation and reprogramming process, leading to beta-cell functional loss [13]. Interestingly, studies involving dieting and bariatric surgery resulted in T2D reversal. These observations gave insights on diabetes etiology after investigating the liver and pancreas during a hypocaloric period. Exposure of the liver to high amounts of triacylglycerol (TAG) or fatty acids, induced increased lipid transportation by very low density lipoprotein (VLDL) towards peripheral tissues, including the pancreas. Chronic exposure to those fatty acids can disturb the normal response mechanism of the pancreatic islets to changes in glucose level [14].

## 3. The Case for Quantifying Beta Cells

A common way to diagnose diabetes consists of measuring the activity of beta cells through glucose metabolism indicators such as plasma glucose, glycated hemoglobin (HbA1c), insulin or C-peptide. These indicators provide information on the beta-cell function (BCF) but poorly reflect the beta-cell state. More importantly, they correlate inaccurately with disease progression, especially in the prediabetic phase, as insidious complications due to chronic hyperglycemia could go undetected for many years. Another hallmark of diabetes, beta-cell mass (BCM), has been suggested as a complementary approach to study disease progression as it behaves differently in the pathophysiology of T1D and T2D, in contrast to BCF [15,16]. However, contrary to BCF measurements, investigation of the BCM is restricted as it requires highly invasive and potentially harmful procedures, such as pancreatic biopsies, making diagnosis and monitoring processes tedious. Given the mystery surrounding beta-cell fate during the progression of diabetes within human patients [17], as well as the high BCM variability between individuals [18], there is a clear need for a precise characterization of changes in the BCM to better understand the physiopathology behind the development of the disease [19]. 

Furthermore, recovery of a functional BCM has been largely described as a viable treatment solution for diabetes [20], meaning that precise quantification and visualization of the BCM would significantly help with designing new therapy strategies by measuring treatment effects and monitoring subsequent changes in the BCM. Islet transplantation, for instance, has been considered for many years as a solution for diabetes reversal, following the loss of insulin producing BCM. Pioneering works by Baker et al. indicated that the liver was an optimal grafting spot for portal vein infusion [21], a method which served as the basis for the now widely accepted Edmonton protocol, which was developed by Shapiro et al. The Edmonton protocol guarantees high successful transplantation rates, while reaching insulin independence with close to normal glycemic control, translating the procedure from an experimental feat to a routine clinical procedure [22]. Excluding auto-transplantations, islet transplantations from donors would, however, require heavy immunosuppressive drugs that would place a heavy burden on the patient and be faced with a widespread shortage of potential donors. 

Several renewable cell sources with the potential to differentiate into beta-cell replacements were explored as alternatives [23], starting with the use of human embryonic stem cells (hEScs). The resulting insulin-producing cells were very similar to endogenous beta cells, but had the critical flaw of a poor response to glucose stimulation [24]. New insights were introduced by Kroon et al. after suggesting in vivo functional maturation of stage 4 pancreatic progenitor cells, previously unattainable from in vitro conditions [25]. Lastly, transplantation of differentiated hEScs through a macroencapsulated delivery system allowed protection of the transplants against the host immune system, while maintaining a proper exchange of nutrients, albeit still allowing the maturation process that was necessary to reach production levels that were close to that of native beta-cell hormone levels [26]. 

Another option is to convert exocrine pancreatic cells into insulin producing cells using transdifferentiation. This technique is based on a reprogramming process via a combined expression of various transcription factors to induce the transition of a differentiated cell into another cell type [27]. Work by Zhou et al. brought to light the role of three critical transcription factors: Ngn3, Pdx1 and Mafa. These three transcription factors are sufficient for in vivo reprogramming of exocrine cells into beta cell-like cells that do not aggregate into islet-like structures, thus are at risk of causing disruption in the normal response to glucose [28].

Regardless of the chosen transplantation method, monitoring transplanted islets remains a hurdle to be overcome, especially during the critical early post-transplantation period that dictates graft survivability [29] as it is particularly sensitive to immunosupressive drugs and inflammation stress. 

Lastly, accurate and rapid evaluation of novel immunomodulatory therapy methods to curb BCM destruction, such as using pancreatic glutamic acid decarboxylase (GAD) autoantigens administration for an improved T-cell immunity reaction [30,31,32], or anti-CD3 monoclonal antibodies treatment to stimulate regulatory T-cells activity [33], is challenging without a direct readout of BCM. Thus, reliable methods for in vivo BCM quantification would be a crucial addition to available clinical endpoints in this area of drug development.

## 4. In Vivo Quantification of Beta-Cell Mass through Positron Emission Tomography

**Figure 1:**
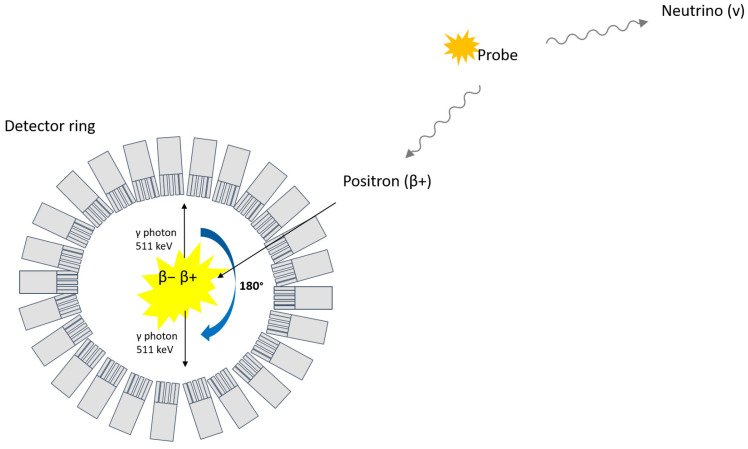
Principle of PET.

Nuclear medicinal imaging techniques using radiation-emitting tracers have been suggested as potential non-invasive tools for the visualization and quantification of BCM. Positron emission tomography (PET), for instance, is based on the detection of two opposite 511 keV photons that follow the annihilation of an electron with a positron emitted from a radioactive probe (Figure 1). 

**Figure 2:**
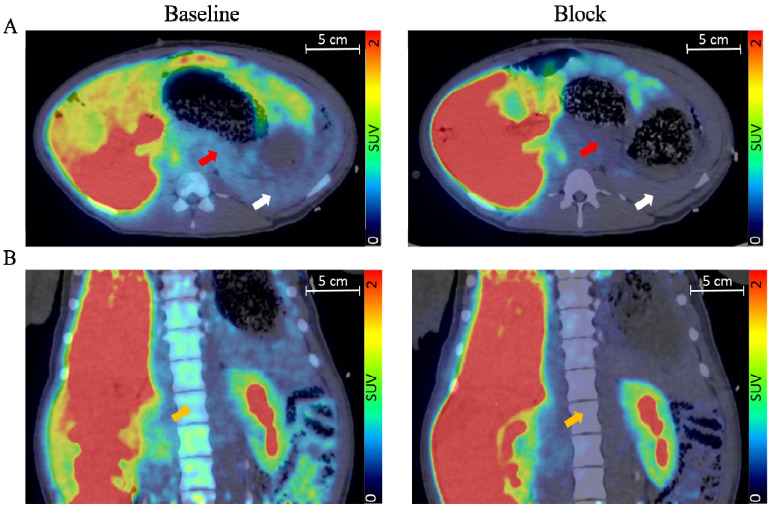
Transversal (**A**) and coronal view (**B**) of fused pig PET/CT scan with the averaged signal from 60–90 min. The red arrow indicates the pancreas, the white arrow indicates the spleen, and the orange arrow indicates bone marrow. Adapted with permission from Cheung et al. [36].

Succinctly, PET possesses higher resolution (~3–5 mm) and better signal quantification compared to other nuclear imaging techniques, such as single photon emission tomography (SPECT) [34]. Both techniques are, however, highly sensitive, meaning low amounts of tracer are enough to allow detection, an important parameter for clinical use to avoid potential adverse pharmacobiological effects. The highly specific uptake of a radioactive tracer within beta cells would usually be represented as being contained in a set of voxels with values reported as concentration (signal/volume) or standardized uptake value (SUV), which can subsequently be represented as hotspots on an image (Figure 2). The signal derived from a virtually perfect tracer for BCM would yield a high specific binding signal coupled with a low background signal that would correlate to a quantitative estimate of the beta-cell density [35].

Although other BCM imaging modalities, such as magnetic resonance imaging (MRI), possess excellent spatial resolution and do not involve any radioactive emission, the MRI signal is not quantifiable in contrast to PET. Moreover, MRI remains highly unspecific and it is not possible to distinguish endocrine and exocrine tissues without the use of potentially toxic paramagnetic contrast agents.

**Figure 3:**
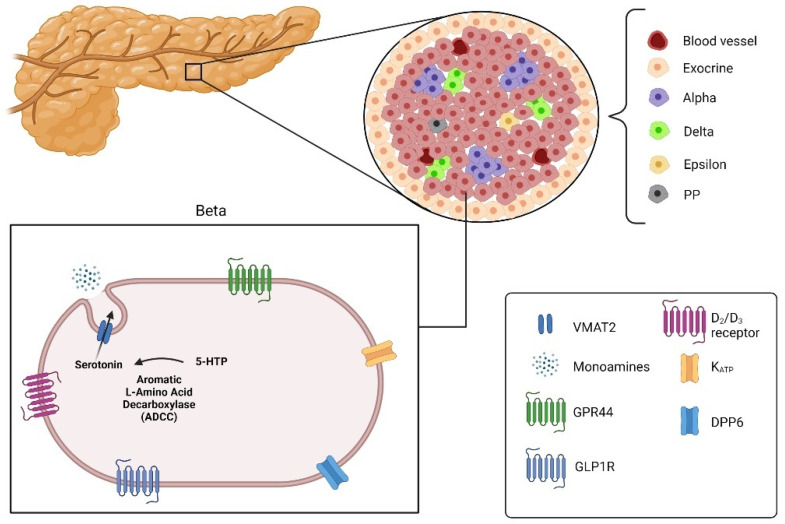
Pancreatic islets divided into alpha, beta, delta, epsilon and pancreatic polypeptide cells (PP) with the targets for imaging beta cells: Vesicular monoamine transporter 2 (VMAT2), G-protein-coupled receptor 44 (GPR44), glucagon-like peptide 1 receptor (GLPIR) and Dipeptidyl peptidase 6 (DPP6) (created with the help of BioRender.com https://biorender.com/ accessed on 31 October 2021).

Imaging the pancreatic islets using PET still faces many challenges, notably the small size of the islets (~20–500 µm), but also its heterogeneous repartition over the pancreas and the non-uniform microarchitecture. Those restrictions thus require the development of highly specific and sensitive imaging targets and probes. Ideally, the generated probe should display a signal many folds higher in endocrine islets compared to exocrine tissues, but also significantly higher than the non-specific signal arising from the surrounding tissues as well as the unbound plasmatic tracer associated with organ perfusion. It is thus a matter of paramount importance to identify a molecular target that is not only selectively expressed on beta cells, but also available for molecular binding to a probe. Several currently studied BCM biomarkers (Figure 3) will be addressed as part of the present review.

### 4.1. Glucagon-Like Peptide 1 Receptor

The glucagon-like peptide 1 receptor (GLP1R) is a therapeutic target for diabetes due to its involvement in insulin release through stimulation of the adenyl cyclase [37], with several GLP1 agonists currently in clinical use. Studies have also showed the role of GLP1R in beta-cell differentiation [38]. Importantly, GLP1R has been reported to be expressed in the human pancreas [39] and confirmed through monoclonal antibody staining [40], leading to extensive studies of the peptide Exendin-4, a GLP1 analogue with better stability, as it is not subject to dipeptidyl peptidase IV degradation [41]. With the increased availability of gallium generators, the radionuclide Gallium-68 became a widely popular PET radionuclide. Moreover, the half-life of Gallium-68 (68 min) is short enough to not induce a heavy patient burden, a highly appreciated characteristic for clinical translation. Boss et al., for instance, showed through human dosimetry that ^68^Ga-NODAGA-Exendin-4 imaging was likely safe for repeated scans in adults and children, ideal for longitudinal studies [42]. Selvaraju et al. demonstrated non-invasive in vivo imaging of rodent and non-human primates using ^68^Ga-DO3A-Exendin4 [43] with a significant signal uptake in healthy rat pancreases compared to streptozotocin (STZ) induced diabetic rats and a well-defined pancreas in non-human primates. Clinical translation of ^68^Ga-DO3A-Exendin4 by Eriksson et al. demonstrated the first non-invasive PET quantification of GLP1R in the pancreas of T2D patients, as well as a simplified scanning protocol with improved reproducibility [44].

In parallel, the increasing affordability of cyclotrons points towards Fluorine-18 as the next logical choice for PET imaging, as it offers improved resolution due to its lower energy range, as well as an ideal working half-life (109 min).^18^F-Exendin 9–39 was first synthesized by Wang et al. and evaluated in rats. PET images showed, however, low signal uptake in the pancreas compared to the surrounding tissues and no difference between a control and STZ-induced diabetic rats, nor in the spontaneous diabetes BioBreeding diabetes-prone (BB-DP) rat strain [45]. Subsequent work by Kimura et al., using various ^18^F-Exendin 9–39 derivatives, showed increased pancreas to organ ratio in addition to lower liver and kidney uptake using ^18^F-FB40-Ex 9–39 in mice [46]. A phase 1 clinical study of ^18^F-Exendin-4 by Fujimoto et al. showed a safe dosimetry profile with no adverse events. The pancreas could be clearly visualized with a pancreatic SUV higher than the surrounding GLP1R non expressing tissues. BCM prediction was however not investigated, warranting further investigations [47]. GLP1R has also been reported as a promising marker for insulinoma [48,49], but will not be the subject of this review. 

Despite the aforementioned promising results, GLP1R as a biomarker for BCM imaging has also several shortcomings. Given the beneficial effect of the GLP1 signaling on insulin secretion and the maintenance of beta cells in response to glucose level [50], GLP1R expression is subject to variation depending on the metabolic state [51], posing a liability in the value of GLP1R as a biomarker for BCM. Reports also showed significant uptake in alpha cells [52] and the exocrine pancreas [53], although this statement is still very much under debate [54]. Lastly, species variations of GLP1R make the lack of reliable animal models difficult for subsequent probe development [55].

### 4.2. Monoamine Receptor

Vesicular monoamine transporter (VMAT) is an active membrane transporter protein playing an important role in the uptake of vesicles containing monoamines, such as dopamine, adrenaline, histamine and serotonin [56]. The expression profile of the two isoforms (VMAT1 and VMAT2) has been studied and reported in tissues such as the central nervous system, the autonomic nervous system and the neuroendocrine cell system. The expression pattern is mostly mutually exclusive in the given tissue, with VMAT2 expressed exclusively in the beta cells of the endocrine pancreas [57]. Dihydrotetrabenazine (DTBZ) is a well-known analog of the FDA-approved tetrabenazine (TBZ), a monoamine depleter drug used for movement disorder treatment [58]. As such, the positron emitter radioligand ^18^F-FP-DTBZ (also known as ^18^F-AV-133) has been used to characterize BCM. Normandin et al. provided early clinical trial data using ^18^F-DTBZ for in vivo T1D with a good correlation to insulin during disease progression, supplied by validation data from kinetic modeling using arterial signal as input functions [59]. Cline et al. showed that in vivo VMAT2 decrease in the whole pancreas (body and tail) was correlated with BCF in long term T2D patients, showing the underlying link between BCM loss and glycemic control deficiency [60]. Results were further supported by Saisho et al., who again demonstrated the correlation between VMAT2 and insulin expression in healthy and diabetic patients; VMAT2-negative but insulin-positive cells could be observed, but this was attributed to the expression polarity of VMAT2 cellular localization [61]. 

A glaring weakness of using VMAT2 as a target for imaging is the noted expression in pancreatic polypeptide (PP) cells of the endocrine pancreas, warranting caution when interpreting results, as the observed signal uptake of ^18^F-FP-DTBZ could possibly be the result of non-specific binding [62]. Lack of an appropriate reference tissue heavily hinders the possibility of estimating the non-specific binding signal. Naganawa et al. came up with a suggestion for quantifying non-specific binding using the low stereospecific affinity inactive enantiomer of ^18^F-FP-DTBZ, by scaling the computed non-specific binding potential spleen value to accurately estimate non-specific signal uptake in the pancreas [63]. Given the overlapping presence of receptors and transporters in neural tissues and endocrine pancreas, Bini et al. cross-referenced currently available brain radioligands with a beta-cell gene atlas before going through a human screening. The process resulted in the carbon-11 dopamin D2/D3 receptor agonist radioligand ^11^C-4-propyl-9-hydroxynaphthoxazine (PHNO) [64]. Follow-up work by the same research group, using ^11^C-PHNO on a cohort of healthy controls and T1D using region comparison kinetic modeling, showed that a short 30 min scan and the SUVR-1 parameter could provide quantitative measurement of the BCM. High amounts of radiometabolites could potentially be a source of confounding signal, but longer scanning time suggested the unlikeliness of the issue; however, it highlighted the importance of a validated reference tissue when using kinetic modeling if arterial blood sampling is to be bypassed [65].

### 4.3. GPR44

Recent proteomics and transcriptomics from Lindskog et al. identified a new transmembrane G-protein-coupled receptor GPR44 (also known as CRTH2, PTGDR2, or CD294) posited as a promising biomarker for BCM monitoring [66]. GPR44 has been reported to be highly expressed in beta cells of the human pancreas or other large animals, such as non-human primates and pigs, but not in the exocrine nor other endocrine tissues of the pancreas [67]. Activation of the GPR44 signaling pathway was assumed to inhibit insulin secretion, but oral administration of a GRP44 inhibitor showed no major impact on insulin secretion in patients with type 2 diabetes [68], albeit demonstrated improvement of islet function under inflammatory and hyperglycemic stress [69]. Inhibition of GPR44 has been well studied in inflammatory processes responsible for allergy and asthma [70], leading to the development of numerous GPR44 antagonists for the treatment of asthma and other allergic diseases, some of them even reaching late clinical phase drug development [71]. Radiolabeled small molecule GPR44 antagonist AZ12204657 with carbon-11 before evaluation in vitro and in vivo by PET, showed great promise regarding visualization and quantification of BCM [72,73]. MK7246 is a GPR44 antagonist originally developed to treat respiratory diseases [74] and is reported to selectively bind to GPR44 in a reversible manner with high affinity and good pharmacokinetic properties [75]. Initial preclinical evaluation of ^11^C-MK7246 by Eriksson et al. and Cheung et al. demonstrated a clear binding of ^11^C-MK7246 to GPR44 through a receptor/ligand binding mechanism, as the signal uptake could be saturated with non-radioactive MK7246. The optimal biodistribution window for ^11^C-MK7246 was discovered to be between 60–90 min after the hepato-biliary excretion of the radiopharmaceutical compound, revealing a well-defined pancreas on the pig images with no spillover from adjacent tissues [36,76]. Furthermore, an important feature of MK7246 is the presence of a fluorine atom which can thus theoretically be labeled with fluorine-18, a radioactive nuclide with lower energy range; thus, allowing greater resolution and will likely be the subject of future studies.

### 4.4. Potassium Channels

ATP-sensitive potassium channels (K_ATP_) have been long discovered within the human pancreatic beta cells [77,78,79] and play a major role in membrane depolarization-induced insulin secretion [80]. The pancreatic K_ATP_ is composed of multiple subunits, among them is the sulfonylurea receptor 1 (SUR1), which contains the binding site for sulfonylrurea ligand [81]. SUR1 has been suggested as potential biomarker for pancreatic islets [82], with many SUR1 targeting selective pharmaceutical small molecules currently available on the market [83]. Among them, the well-known FDA approved drug glibenclamide (also called glyburide) for T2D treatment [84], a SUR1 selective antagonist that has been extensively studied as potential surface marker for pancreatic endocrine cells with promising results [85,86]. However, Schneider et al. raised the question as to whether glibenclamide and its fluorinated derivatives are suitable for BCM imaging, despite showing promising SUR1 binding potential and affinity. Indeed, a poor signal-to-noise ratio, due to plasma protein binding and possibly attributed to poor lipophilicity of the molecule, has been reported [87]. The same group suggested, however, a glibenclamide glucose-conjugate as a substitute to improve hydrophilicity and a better biodistribution pattern [88].

### 4.5. Serotonin Synthesis Pathway

Serotonin is best known as a neurotransmitter for its role in mood regulation. However, pancreatic beta cells have been showed to be able to synthesize and secrete serotonin as well, most likely due to a shared embryogenesis process between neural and pancreatic endocrine tissues [89], with detailed reports mentioned in the literature describing the role of beta-cell intracellular serotonin production and secretion on glycemic control [90,91]. The uptake pattern of serotonin’s direct precursor 5-Hydroxytryptophan (5-HTP), is characterized by fast tissue uptake followed by fast wash out, unless it is further metabolized following the classical intracellular serotonin synthesis pathway [92]. This implies that cellular retention of 5-HTP requires serotonin synthesis machinery present in the islet cells, but absent in the exocrine cells [93]. Carbon-11 labeled ^11^C-5-HTP has therefore been explored as a potential non-invasive in vivo tracer for quantitative variation in pancreatic islets, with results indicating a clear correlation between ^11^C-5-HTP cellular retention and the number of pancreatic islets [94,95]. A longitudinal assessment of pancreatic islets in T1D patients using ^11^C-5-HTP by Espes et al. gave new insights on endocrine pancreas volume variations during T1D disease progression right after disease onset [96]. Considering the ^11^C-5-HTP uptake signal as representative of the endocrine pancreas, the group observed a poor correlation between pancreatic volume and BCF measured through C-peptide and HbA1c levels. The finding goes, however, against the mainstream postulate suggesting major beta-cell destruction within the 2-year period following diagnosis of T1D. Although the signal from ^11^C-5-HTP is unlikely to be from exocrine tissues, signal uptake is an approximation of BCM as the signal represents overall endocrine pancreatic islets composed by a majority of beta cells. Further studies using beta-cell-specific PET tracers are thus needed to enable direct assessment of BCM.

### 4.6. Dipeptidyl Peptidase 6

Dipeptidyl peptidase 6 (DPP6) is a protein discovered through transcriptomic studies of the human BCM by Eizirik et al. as a membrane surface marker [97]. Subsequently, Balhuizen et al. developed a nanobody, a small peptide (~13 kDa) derivative from homodimeric heavy chain antibodies, and managed to image insulin-producing EndoC-βH1 transplanted mice using SPECT/CT with ^99m^Tc-4hD29 [98]. Demine et al. later on developed a camelid nanobody 4hD29, labelled with gallium-68 that was suitable for PET imaging by conjugation with NCS-NOTA. Results showed good uptake in nude mice transplanted with human pancreatic islets grafts and were validated as a proof of concept using Kelly cells, a DPP6 positive neuronal cell line [99]. It is important to emphasize that the above mentioned studies were all based on imaging a high number of transplanted cells in a relatively small volume, which would possibly not be replicable in actual endocrine pancreatic cells dispatched heterogeneously over the pancreas. Another limitation of targeting DPP6 is the expression within beta and alpha cells. On the other hand, reports suggest an overall decrease in alpha-cell populations and function in T1D patients [100], making 4hD29 still a valuable BCM targeting probe.

## 5. Conclusions

Visualization and quantification of BCM is still a much-debated topic with strongly divided views. Alavi and Werner, for instance, describe the limitation of PET and the structural requirement of the imaging target, along with the aim of BCM imaging, as “futile”. Indeed, the feasibility of PET pancreatic imaging is heavily limited due to several restrictions that could be summarized as: (1) biological size of beta cells that are much below the resolution obtainable by current PET scanners; (2) variations in the pancreatic volumes in T1D and T2D patients that would invalidate BCM quantification as a signal to volume ratio; (3) a necessity to clearly distinguish specific binding signal from the non-specific signal that could arise from the vastly predominant exocrine pancreatic tissue; (4) high variability in blockade studies with the presence of residual uptake in diabetes subjects; and (5) lack of promising results from ex vivo autoradiography results [101].

On the other hand, Gotthardt et al. argue that spatial resolution is not the end goal for BCM imaging, as the high sensitivity of PET technology would instead allow the detection of a highly specific signal from the beta-cell population from the metabolic trapping of the tracer, in contrast to a low background signal from the exocrine tissue. The idea would be to rely on a proof of concept, highly specific tracer binding to pancreatic islets and to quantify the large, relative to voxel size, organ of interest (i.e., the pancreas) [102]. 

As progress in the PET imaging modality is highly restricted by technological advance in the field, future research should be focused on large in silico screenings for novel BCM restricted biomarkers, in addition to computational design of new imaging probes with strong molecular binding affinities, biodistribution kinetics and adequate systemic clearance properties. Regardless, progress in the field of BCM imaging has seen huge advancements in recent years, highlighted by the many tracers explored in the field (Table 1), with increasing probability to eventually reach the human clinical stage.

## Figures and Tables

**Table 1:** List of targets for PET BCM imaging and the corresponding available probes.

Target	Target Limitations	Radioactive Probe	Tested Models	Reference
*GLP1R*	Expressed in ductal and exocrine tissues Species variations Expression level depend on metabolic state	68Ga-NODAGA-Exendin-4 68Ga-DO3A-Exendin4 18F-FB40-Ex 9-39 18F-FB(ePEG12)12-exendin-4	In Vivo (human) In Vivo (rodent, non human p, human) In Vivo (mice) In Vivo (human)	Boss et al. [42] Selvaraju et al. [43]; Eriksson et al. [44] Wang et al. [45]; Kimura et al. [46] Fujimoto et al. [47]
*VMAT2*	Expression in pancreatic polypeptide cells Lack of reference tissue	18F-DTBZ	In Vivo (human)	Normandin et al. [59]; Cline et al. [60]; Naganawa et al. [63]
Dopamine receptor	Rapid metabolism of propyl-hydroxynaphthoxazine leading to limited tracer uptake Difficulties in differentiating specific and non specific signal	11C-PHNO	In Vivo (human)	Bini et al. [64]
*GPR44*	Lack of reliable in vivo model expressing G-protein-coupled receptor 44 Not yet tested in human	11C-AZ12204657 11C-MK7246	In Vitro; In Vivo In Vitro (CHO-K1 cells); In Vivo (Pig)	Jahan et al. [72]; Eriksson et al. [73] Eriksson et al. [76]; Cheung et al. [36]
*SUR1*	Poor signal-to-noise ratio	18F-Glibenclamide	In Vitro (rat islets); In Vivo (human)	Schneider et al. [87,88]
Serotonin	Not a membrane receptor so more indicative of beta-cell function than beta-cell mass	11C-5-HTP	In Vivo human	Eriksson et al. [92,94]; Lubberink et al. [95]; Espes et al. [96]
*DPP6*	Expression within alpha-cells Not yet tested in human	68Ga-4hD29	In Vivo (transplanted grafts EndoC-βH1 and human islets into nude mice)	Balhuizen et al. [98]; Demine et al. [99]

## Data Availability

Not applicable.
